# Applying Van den Akker’s Spider Web Model to curriculum design in medical education: a practical guide

**DOI:** 10.3389/fmed.2026.1713404

**Published:** 2026-04-24

**Authors:** Güneş Korkmaz

**Affiliations:** Department of Medical Education, Faculty of Medicine, Istanbul Medeniyet University, İstanbul, Türkiye

**Keywords:** conceptual framework, curriculum design, curriculum development, medical education, Spider Web Model

## Abstract

Contemporary medical education requires curriculum models that ensure coherence, alignment, and adaptability to rapid technological and societal changes. Van den Akker’s Curricular Spider Web Model, a well-established framework in general education, has not yet been systematically applied in medical education. This conceptual paper, which employs conceptual framework analysis approach, presents the model’s application as a guiding framework for curriculum design in undergraduate medical education. Rather than proposing a new theoretical model, this paper illustrates how the Spider Web can complement widely used frameworks such as Tyler’s objectives-based model, Harden’s SPICES framework, and Kern’s Six-Step Approach. Sample applications of the model are presented at nano, micro, and meso levels, with specific attention to the integration of artificial intelligence in the curriculum. The applications indicate that the Spider Web Model provides a comprehensive and flexible structure that can guide curriculum development in medical education by promoting coherence, alignment, and stakeholder engagement. The proposed framework offers practical guidance for curriculum designers, medical educators, and academic leaders, supporting the alignment of curriculum components across nano, micro, and meso levels. It is particularly useful in contexts requiring integration of emerging domains, such as artificial intelligence, into existing curricula, facilitating coherent and adaptable curriculum development Future empirical research is needed to examine its implementation and impact on educational outcomes.

## 1 Introduction

The primary goal of medical education is to prepare well-informed, skilled physicians who prioritize patient care over personal gain and are committed to continuously advancing their expertise throughout their careers (1). While medical education encompasses undergraduate, postgraduate, and continuing professional development phases, this paper primarily focuses on the undergraduate medical education context, where foundational knowledge, skills, and professional values are first developed. To achieve this, medical schools must provide learners with a strong foundation of scientific knowledge, clinical skills, and professional values, while fostering commitment to ethical practice and lifelong learning. A well-designed curriculum plays a central role in realizing this goal, as it provides the structured framework through which medical schools can instill the necessary knowledge, skills, and values in future physicians. The curriculum serves as the cornerstone that supports all other aspects of medical education and can be seen as the pathway, tool or guide to achieving success. Similar to a well-constructed road, an effective medical curriculum enables all components of medical education to operate in coordination and harmony (2, 3). In other words, curriculum provides policy makers and medical educators with a roadmap for addressing various components such as setting learning goals, creating content, selecting instructional strategies, choosing methods and techniques for learning and teaching activities, determining appropriate materials and resources, arranging the learning environment, and planning how learning will be assessed (4). Therefore, curriculum development in medical education has been an important topic of discussion.

Especially after Abraham Flexner’s Report was published in 1910, the nature and process of medical education had to be transformed. The reasons for this transformation were mainly *overproduction of poorly trained doctors disregarding public welfare, commercialized medical schools, low-quality education from underfunded schools, the existence of many unnecessary and inadequate medical schools, and the need for teaching hospitals which open their wards for teaching* (5). Since then, all over the world, a critical transformation has begun in medical education by closing poor schools, raising standards, integrating clinical teaching with research, and emphasizing full-time faculty in university hospitals (6–10). Building on this scientific and structured foundation established after the Flexner report, medical schools started applying different educational models such as problem-based learning (PBL), competency-based education (CBE), and integrated model (11–14). These models have been most widely implemented in undergraduate medical curricula, particularly during the preclinical and early clinical phases, where medical educators aim to foster integration between basic and clinical sciences and to develop core professional competencies.

### 1.1 Common curriculum models in medical education

To guide educators and institutions in designing effective, relevant, and learner-centered medical curricula, a variety of theories, strategies, approaches, and models have been developed. Among these, three influential models widely used in medical education are Tyler’s curriculum development model, Harden’s SPICES model, and Kern’s Six-Step Approach to curriculum development. These models have provided frameworks that guide curriculum developers in aligning educational goals, content, instructional methods, measurement, assessment and evaluation strategies. While these curriculum development approaches and models have provided valuable foundations, they are not without limitations in addressing the evolving needs of modern medical education (15–17) considering contemporary medical curricula must contend with increasingly complex healthcare systems, rapidly expanding biomedical knowledge, advances in educational technology, and shifting societal expectations from physicians (18).

Ralph Tyler’s curriculum development model is one of the earliest systematic approaches to curriculum development which is based on these four fundamental questions: *What educational objectives should an educational institution seek to attain?*, *What educational experiences can be provided to attain these purposes?*, *How can these educational experiences be effectively organized?* and *How can we determine whether these purposes are being attained*? In this sense, Tyler’s model focuses on defining clear objectives, aligning content and methods with these objectives, and evaluating outcomes, and the primary focus of this linear model is on the significance of clearly predetermined objectives (19). However, this model has been criticized for its lack of flexibility required for integrating competencies such as professionalism, communication, interprofessional collaboration, and systems-based practice (17), and as its prescriptive, objective-centered approach does not align well with contemporary demands for flexible, learner-centered, and competency-based curricula (20).

Similarly, Harden’s SPICES model has encouraged innovation through strategies like learner-centered and problem-based approaches (21). Rather than a curriculum development model, the SPICES model was introduced as a strategic framework to shift traditional medical curricula. SPICES is an acronym for student-centered (rather than teacher-centered learning), problem-based (instead of information-based teaching), integrated (as opposed to discipline-based structure), community-based (rather than hospital-based education), electives (instead of standard programs), and systematic (rather than opportunistic/apprenticeship models) (17, 22). In addition, Harden developed another framework for curriculum integration through integration ladder which consisted of 11 steps describing the integration degree of a curriculum on a continuum ranging from isolation (no integration) to transdisciplinary (fully integrated curriculum) (23). The 11-step integration ladder is seen as a useful tool as it supports thoughtful curriculum development by offering a clear range of options for blending integrated and subject-based approaches (24), and also a tool for curriculum development and evaluation (25). As the integration ladder advances to a higher level, the need grows for a centralized organizational structure, wider involvement of faculty and subject matter experts in planning, and robust channels of communication in curriculum planning (26). By outlining diverse strategies, it guides curriculum designers and educators to collaboratively explore and determine the most suitable forms and levels of integration for their educational programs (27, 28).

Another approach to curriculum development in medical education is Kern’s Six-Step Approach which systematize the process for curriculum design including the following steps: *problem identification and general needs assessment, targeted needs assessment, goals and objectives, educational strategies, implementation, and evaluation and feedback* (29, 30). According to Kern, curriculum development is not a strictly linear process but rather a dynamic and interactive one, where multiple steps often occur simultaneously and influence each other (31). For example, implementation activities may begin as early as the general needs assessment phase, and resource limitations can shape both the objectives and evaluation scope. Similarly, evaluation efforts can lead to revisions in objectives or inform future needs assessments. Additionally, time constraints or existing curricula may prompt the development of goals, methods, and implementation strategies before formal problem identification, with early steps later used to refine rather than create a curriculum from scratch (30). Compared to Tyler’s and Harden’s frameworks, Kern’s six-step approach offers a more practical and iterative model for curriculum development in medical education. However, although it provides clear steps for constructing a curriculum or educational program at a module or course level, it offers less explicit guidance on system-wide issues such as aligning curricula across multiple years or disciplines, pre-clinical-clinical year integration (vertical-horizontal), managing institutional change, or integrating new technologies and educational innovations across a program.

Contemporary medical education faces increasingly complex demands, including accreditation requirements, competency-based education, Entrustable Professional Activities (EPAs), interprofessional education (IPE), artificial intelligence integration, and a heightened focus on equity, diversity, and social accountability (18, 32–38). These multifaceted challenges necessitate curriculum development processes that are dynamic, integrated, and system-oriented, and the evolving landscape of medical education, shaped by rapid technological advancements and shifting societal expectations, requires ongoing adaptation in curriculum design approaches (39, 40). In response, medical educators and policy makers must reconceptualize curriculum development as a strategic, institution-wide endeavor that extends beyond isolated innovations and emphasizes coherence and interconnectedness across all elements of curriculum design (41–43).

To achieve better and clearer application of models and complementary tools in curriculum design, medical teachers must take role as change agents in curriculum planning and development (44). However, despite the availability of guiding models and approaches of curriculum design, many educators and policy makers lack a clear and shared understanding of their purpose and application. This gap may be attributed to the inherent complexity and interdisciplinary nature of curriculum development, variability across educational contexts, and limited exposure to formal training in curriculum theory. Consequently, the concept of curriculum and existing models often remain abstract for those involved in curriculum design and implementation. In this context, this paper aims to explore the applicability of van den Akker’s Curricular Spider Web Model in undergraduate medical education. By framing this discussion within a conceptual framework analysis, the study contributes to the theoretical advancement of curriculum development in health professions education. Therefore, this study illustrates how van den Akker’s model can be applied in undergraduate medical education and highlights its potential complementarity with established frameworks and curriculum development models widely used in undergraduate medical education.

### 1.2 Introducing Van den Akker’s curricular Spider Web curriculum model

**Figure 1:**
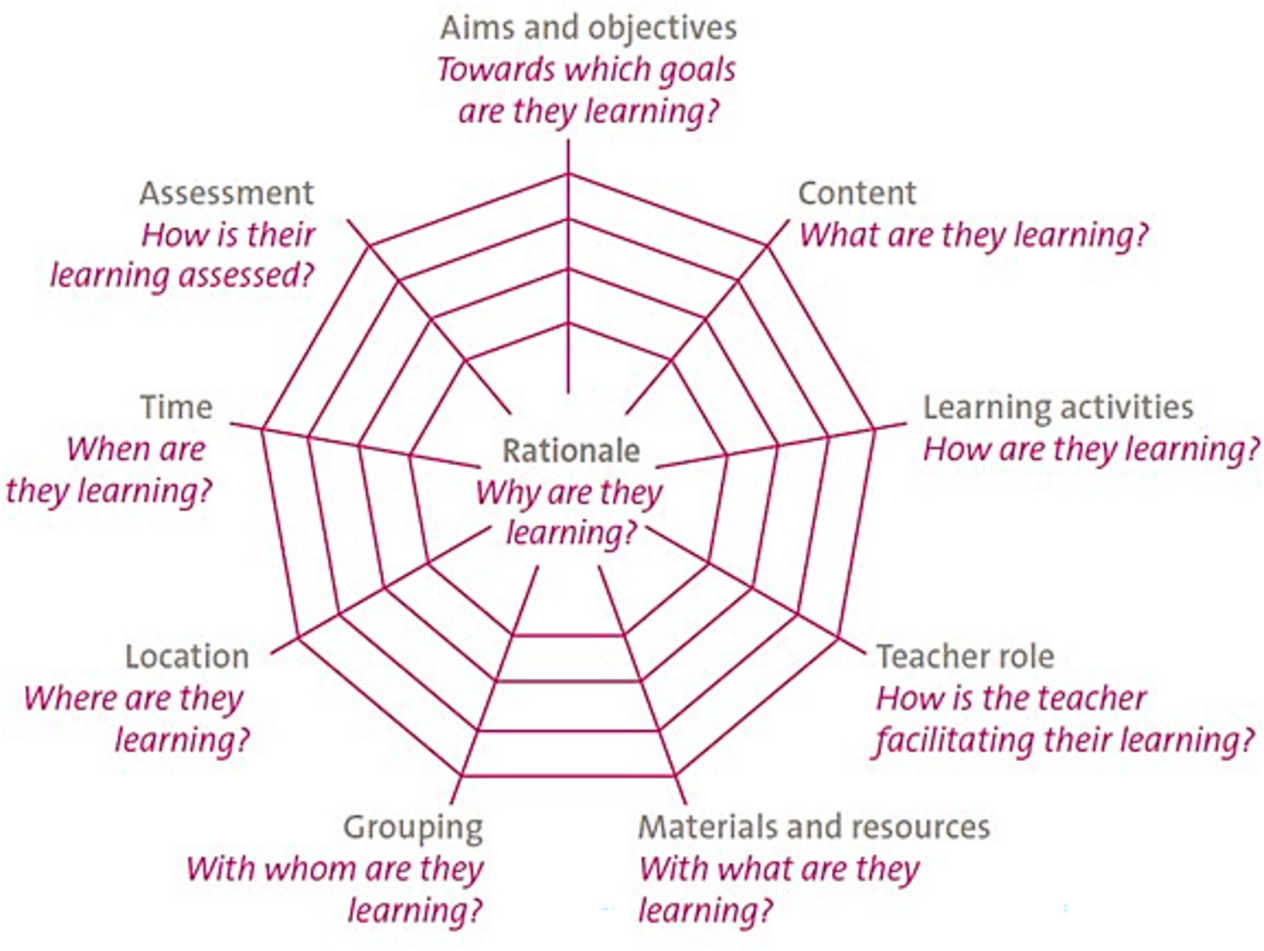
Curricular spider’s web [Source: Van den Akker (45)].

Van den Akker’s Spider Web Model offers a compelling alternative to the curriculum development models introduced before. It uses the spider web metaphor to highlight the fragile and interdependent nature of curriculum components. Rather than focusing on the three major planning elements (content, purpose and organization of learning), van den Akker states a more elaborate list of components is needed to address 10 specific questions about the planning of student learning (45). Originally created to be used in general education, the model conceptualizes curriculum as a web of 10 interconnected elements: rationale, aims and objectives, content, learning activities, teacher role, materials and resources, grouping, location, time, and assessment. Nine components radiate from the central component rationale. The strength of the curriculum, much like a spider’s web, depends on the balance and integrity of these elements (45). The visual description of this model is depicted in Figure 1.

As can be seen in Figure 1, the rationale acts as the central anchor in curriculum design, interlinking all other components, and all components should align with the rationale or vision stated in the curriculum. The rationale of a curriculum is typically shaped by the answers to the following questions about knowledge, society and learners (45):

- What foundational academic and cultural content is considered crucial for students’ learning and future growth? (Knowledge),
- What topics and challenges should be addressed based on current societal trends and needs? (Society),
- What aspects are most important for supporting learning, considering the personal interests and educational needs of the students? (Learner).

To shape this rationale, timely and authentic involvement of all relevant stakeholders is needed. From the enactment perspective, teachers and learners are the most important stakeholders that together create their own curriculum rationale, which meets the real needs between ideals and implementation (45). Similarly, in medical education, rationale serves as the foundation of the curriculum, reflecting the overarching mission to train competent, ethical, and socially responsive physicians. It aligns the rationale or vision with societal health needs, scientific advancements, and professional expectations (46). The rationale in medical education curriculum is designed based on national and international competency frameworks such as WFME standards and CanMEDS framework (47, 48).

- *Aims and objectives* describes towards which goals students are learning. This does not mean the teaching objectives which are determined by the teachers or curriculum makers, but the learning outcomes defined in terms of competences, and performance standards (49). In medical education, what learners are expected to achieve by the end of their education and training is described as competencies, and competency-based medical education (50) is the most common strategy used to design medical curriculum (51). In line with the competences, aims and objectives are designed at program and course levels.
- *Content* encompasses the knowledge, attitude and skills that learners need to acquire, and in the model, this component addresses the question what students are intended to learn. In medical education, the content of curriculum is intended to combine basic sciences and clinical medicine, and is organized around organ systems, themes, or real patient cases. The most commonly used content organization approaches in medical curricula are the modular, spiral (52, 53), inquiry-based, core curriculum approach (54, 55). After the content is determined and organized, it is necessary to prepare a matrix, which shows the relationship between the learning outcomes and the content, which serves as a guide in determining assessment methods and techniques (56).
- *Learning activities* in the model is about how students learn. In this process, the planning focuses on how teaching and learning will be designed in order to help learners acquire the expected knowledge, skills, attitudes, and values (57). In medical education, activities are designed as lectures, problem-based learning (PBL), case-based learning (CBL), team-based learning (TBL), flipped learning, clinical simulations, skills labs, case discussions, and clinical rotations. These activities are designed to promote active learning, critical thinking, and the application of theoretical knowledge to clinical scenarios. Effective learning activities often integrate basic and clinical sciences to foster deep understanding.
- *Teacher Role* is another important component in the model which addresses the question about how the teacher facilitates students’ learning. As can be seen from the question itself, the teacher’s role is not to teach but facilitate learning. According to Harden and Crosby, a good teacher is more than a lecturer, and eight roles of the teachers in healthcare professions are *information provider and coach*, *facilitator of learning and mentor*, *curriculum developer and implementer*, *assessor and diagnostician*, *role model*, *manager and leader*, *scholar and researcher*, and *professional* (58).
- The component *materials and resources* deals with the question with what the students are learning. In medical education, materials and resources include textbooks, digital tools, clinical guidelines, anatomy models, e-learning platforms, simulation equipment, digital escape rooms and many more (59–61). And currently, there are studies that suggest medical students use artificial intelligence as a learning tool (62, 63).
- *Grouping* is about with whom the students are learning. Medical students may learn individually, in small groups (e.g., during CBL, PBL, TBL sessions or clinical rotations), or as part of a larger group (64, 65). Grouping strategies should promote collaborative learning, peer learning and teaching, while also providing individualized learning opportunities within the principles of self-directed and self-regulated learning (66, 67). Another important issue in grouping is interprofessional education opportunities for medical students in which they will learn and act together as team members (68, 69), and this should be integrated into undergraduate medical curriculum starting from the early years of medical education to enhance their teamwork and communication skills.
- *Location* addresses the question about where students are learning. Learning in medical education takes place across diverse settings, including lecture halls, simulation labs, hospitals, community health centers, and online platforms such as learning management systems (LMS) (70, 71). As medical education aims to integrate pre-clinical and clinical periods, decision-making on the learning environment is crucial.
- *Time* management in the medical curriculum involves allocating appropriate time for pre-clinical and clinical education phases and ensuring longitudinal integration of topics related to behavioral and social sciences like communication and professionalism. Curriculum planners must also consider student workload and time needed for reflection, self-directed learning, and rest (72, 73).
- *Assessment* in the model deals with how students’ learning is assessed. This question is very important in medical education as students’ progress should be measured using both formative and summative assessment methods to ensure their readiness for clinical practice. This measurement includes a variety of assessment methods and techniques such as written and oral exams, Direct Observation of Procedural Skills (DOPS), Objective Structured Clinical Examinations (OSCE), Mini-Clinical Evaluation Exercise (Mini-CEX), workplace-based assessments, portfolios, etc. As the assessment methods are diverse in medical education, these strategies should be carefully planned to align with learning objectives and support both formative feedback and summative decisions about competence (51). In other words, the learning outcomes should be evident in the assessment (constructive alignment) (74).

## 2 Methods

This paper adopts the principles of conceptual framework analysis (CFA) (75) to explore the applicability of van den Akker’s Spider Web Model to curriculum design in undergraduate medical education. CFA was chosen as an analysis method for its suitability in synthesizing theoretical constructs from diverse sources and constructing a coherent, context-specific conceptual framework. CFA was conducted in eight phases (75), as outlined below:

- Phase 1: Mapping the selected data sources: Relevant literature on curriculum development models, frameworks in medical education, and van den Akker’s model were systematically reviewed. Search was conducted using PubMed, ERIC, and Google Scholar with keywords including “curriculum development,” “curriculum model,” curriculum development model,” “medical curriculum design,” and “medical curriculum development.” Literature from 2010 to 2025 was considered. Inclusion criteria was peer-reviewed articles related to curriculum design and development in medical education. The articles which were written in English and Turkish were selected for inclusion. The ones which are not within the scope of medical education were excluded. As this study is not a systematic review or meta-analysis, no further information was provided about these articles in tables.
- Phase 2: Extensive reading and categorizing of the selected data: Selected materials were read thoroughly, categorized by relevance and importance. Most used curriculum development models in medical education were found to be Tyler, Harden, and Kern’s. Therefore, these studies were identified, read and analyzed extensively.
- Phase 3: Identifying and naming concepts: Key concepts were inductively derived from literature. Original components from van den Akker’s model (e.g., goals, content, learning activities) were analyzed to be in line with the medical education context.
- Phase 4: Deconstructing and categorizing concepts: Each concept in the model was analyzed in terms of its definition, assumptions, and role in curriculum development.
- Phase 5: Integrating Concepts: Overlapping concepts were merged to reduce redundancy and strengthen clarity.
- Phase 6: Synthesis, resynthesis, and making it all make sense: The refined concepts were integrated into van den Akker’s Spider Web Model.
- Phase 7: Validating the conceptual framework: The emerging sample frameworks (nano, micro, meso levels) were shared with medical educators and curriculum specialists for feedback. This phase ensured that the frameworks were both technically feasible and pedagogically sound. The feedback informed the final refinement of the sample frameworks, confirming their practical relevance for medical curriculum. Feedback was obtained from four medical educators (three associate professors of medical education, one curriculum and accreditation committee member). Feedback was collected via structured face-to-face and online meetings focusing on feasibility and clarity of nano/micro/meso examples. In these meetings, each sample framework for nano, micro and meso levels was accompanied by structured feedback columns including the categories “appropriate,” “not appropriate” (with justification), and “suggested revision.” This format enabled a criterion-based and comparable assessment of each component of the framework.
- Phase 8: Rethinking the conceptual framework: Based on feedback from medical educators and specialists, the sample framework tables were reviewed, synthesised, and iteratively revised accordingly. This process ensured that the final framework was both conceptually robust and practically applicable within medical curriculum design.

## 3 Results

### 3.1 Sample curriculum frameworks at nano, micro, and meso levels

Using van den Akker’s Spider Web Model, the sample frameworks were developed to illustrate how artificial intelligence (AI) can be integrated into undergraduate medical education curricula at different levels. Driven by the urgent need to align medical education with the ongoing digital transformation, AI was chosen as the main theme in these sample frameworks intentionally and strategically. Furthermore, medical schools around the world are actively exploring ways to integrate AI into their curricula to prepare future physicians for these changes. Thus, it is believed that these sample frameworks, which are designed using van den Akker’s Spider Web Model, will provide a structured and pedagogically coherent approach for integrating AI across different levels of the undergraduate medical education curriculum. In other words, in this study, AI was not selected as a curricular goal or skill but as a means to foster higher-order cognitive and professional skills rather than as an isolated area of expertise. In addition, AI was chosen to be explained using the undergraduate level, because early exposure to AI concepts helps students understand how emerging technologies influence professional skills, and as it is easier for readers to understand how to use the model at nano, micro and meso levels.

The mappings to EPAs and CanMEDS roles presented in Tables 1–3 are intended as illustrative rather than prescriptive. They were developed using a simple set of decision rules. First, Spider Web elements that define the overall purpose or vision (e.g., rationale, aims and objectives) were aligned with high-level competencies such as the CanMEDS Scholar and Health Advocate roles. Second, elements that focus on processes of learning (e.g., activities, grouping, and teacher role) were linked to EPAs and competencies emphasizing collaboration, communication, and reasoning; and finally, the elements that address evaluation (e.g., assessment) were mapped to roles and EPAs involving judgment of competence. The mappings were iteratively refined through feedback from curriculum experts to ensure conceptual coherence. They should be interpreted as context-specific illustrations rather than validated correspondences, and medical educators and curriculum makers are encouraged to adapt them according to local needs and regulatory frameworks.

**Table 1:** Sample framework for meso-level curriculum design.

Spider Web Model components	Explanations for the questions in the Spider Web Model Components	Examples of implementation	Links to competency frameworks
Rationale *(Why are they learning?)*	To prepare future physicians for AI-augmented healthcare and equip them with digital health competencies	AI strategy integrated into institutional mission and vision, international and national health education strategies referenced	CanMEDS (Scholar, Medical Expert)*, AAMC EPA 13 (Technology Use)**
Aims and Objectives *(Towards which goals are they learning?)*	Ensure all graduates demonstrate AI literacy, ethical reasoning, and the ability to work with interprofessional teams using AI tools	Program-level learning outcomes including AI competencies (Each year should include AI related competences in modules and courses)	CanMEDS (Health Advocate)*, AAMC EPA 9 (Team Collaboration)**
Content (*What are they learning?)*	Specify core AI concepts, digital health policy, data ethics, clinical applications, bias and equity, machine learning fundamentals	Courses on machine learning, data privacy, clinical applications of AI	CanMEDS (Scholar, Medical Expert)*
Learning activities *(How are they learning?)*	Provide diverse, active, and experiential learning approaches to develop competencies by organizing university-wide AI literacy seminars, digital electives, research on AI in medicine, etc.	AI literacy seminars, virtual simulations, interdisciplinary projects, serious games, and AI tool critique assignments	CanMEDS (Scholar, Leader)*
Teacher role *(How is the teacher facilitating their learning?)*	Redefine educator responsibilities as facilitator, mentor, AI leader, interdisciplinary collaborator, innovation enabler, curriculum developer	Faculty development sessions on AI and digital pedagogy, interdisciplinary teaching, educator self-assessments	CanMEDS (Leader)*, AAMC, AACOM, ACGME *** Core Competencies (Interpersonal and communication)
Materials and resources *(With what are they learning?)*	Leverage technology-enhanced learning environments to support digital skill development	AI labs, simulations, AI-supported LMS platforms that provide feedback for individuals, open datasets, and analytics dashboards	AAMC, AACOM, ACGME*** (Digital literacy competencies)
Grouping *(With whom are they learning?)*	Foster interdisciplinary and interprofessional collaboration with interfaculty projects with nursing, data science, engineering, and ethics departments	Joint team projects with other faculties and departments, learning in interdisciplinary teams	WHO Interprofessional Competency Framework****, CanMEDS (Collaborator)* EPA 9**
Location *(Where are they learning?)*	A blended learning ecosystem combining digital and physical spaces	Cross-campus library, clinical skills center, simulation labs, digital labs, serious games-escape rooms	CanMEDS* (Professional workplace settings)
Time *(When are they learning?)*	Spread throughout the 6-year curriculum (the load and emphasis on AI increases in clinical years)	Curriculum mapping that shows the alignment across preclinical-clinical years	AAMC, AACOM, ACGME*** Foundational Competencies for Undergraduate Medical Education
Assessment *(How is their learning assessed?)*	Measure learning outcomes related to AI competencies through formative and summative assessments using Miller’s pyramid	Digital portfolios, practical exams, reflective assessments, project outcomes	CanMEDS (Scholar: Performance Assessment)*, EPA7** (Form clinical questions and retrieve evidence to advance patient care)

*CanMEDS Frameworks (76).

**Amiel et al., AAMC (77).

***AAMC, AACOM, ACGME (78).

****WHO (79).

**Table 2:** Sample framework for micro-level curriculum design (nervous system module).

Spider Web Model components	Explanations for the questions in the Spider Web Model components	Examples of implementation	Links to competency frameworks
Rationale *(Why are they learning?)*	Understand how AI is applied in neurology (e.g., brain imaging interpretation), enhance pattern recognition and clinical reasoning	Module objectives include AI applications, student feedback on perceived relevance of AI in neurology	CanMEDS (Medical Expert)* (Clinical Reasoning), AAMC EPA 1,7**
Aims and objectives *(Towards which goals are they learning?)*	Ensure students can critically evaluate AI applications in neurology, interpret AI-supported diagnostic tools, and identify ethical concerns	Learning outcomes aligned with module objectives and digital health competencies	AAMC EPA 2,4**, CanMEDS (Scholar)*
Content (*What are they learning?)*	AI in neuroimaging, EEG analysis, neural networks in computational neuroscience, ethics of brain data use	Curriculum map integration with anatomy, histology, and physiology content	AAMC, AACOM, ACGME (Practice-Based Learning and Improvement)***
Learning activities *(How are they learning?)*	Case-based learning activities, discussions, AI-driven chat Simulation, clinical vignettes with AI-supported diagnosis, etc.	Student engagement logs, structured observation of diagnostic simulations	CanMEDS (Scholar)*, AAMC EPA 2,3**
Teacher role *(How is the teacher facilitating their learning?)*	Guide for interpreting AI outputs, facilitator of clinical reasoning using digital tools	Faculty members should be trained in AI literacy and facilitation	CanMEDS (Leader)*
Materials and resources *(With what are they learning?)*	AI-enabled 3D brain models, virtual patient platforms, AI-enhanced neuroanatomy atlases	Usage statistics from LMS, resource checklist for educators	AAMC EPA 13**, CanMEDS (Scholar)*
Grouping *(With whom are they learning?)*	Small teams for diagnostic simulation, groups for AI debate	Team-based assessment rubrics, activity reports	AAMC EPA 7**
Location *(Where are they learning?)*	Simulation centers, digital anatomy labs, hybrid classrooms, online platforms	Hybrid Learning Platforms, LMS	CanMEDS (Professional)*, CBME delivery contexts
Time *(When are they learning?)*	AI integration across the module	Mapped into the weekly teaching plan	AAMC, AACOM, ACGME (Individualized learning and flexibility)***
Assessment *(How is their learning assessed?)*	Online quizzes analyzing AI outputs, short reflective essays, serious games like escape rooms	Assessment reports, rubric-aligned feedback, learner analytics, AI tools that assess diagnostic reasoning	CanMEDS (Scholar)*, AAMC EPA 12**

*CanMEDS Frameworks (76).

**Amiel et al., AAMC (77).

***AAMC, AACOM, ACGME (78).

****WHO (79).

**Table 3:** Sample framework for nano-level curriculum design.

Spider Web Model components	Explanations for the questions in the Spider Web Model components	Examples of Implementation	Links to competency frameworks
Rationale *(Why are they learning?)*	Address ethical challenges of AI in healthcare, such as bias, transparency, and accountability	Stated in course guide, alignment with institutional digital ethics goals	CanMEDS (Professional)*(Health Advocate- Social accountability of physicians), AAMC Core EPAs on ethics**
Aims and objectives *(Towards which goals are they learning?)*	Develop students’ ability to reason about the ethical use of AI in medical decision-making, and articulate arguments about fairness, privacy, responsibility and accountability	Learning objectives mapped to national and international ethics standards (e.g., Identify ethical dilemmas in AI use, Apply ethical principles autonomy, justice, non-maleficence, beneficence)	AAMC EPA 1,9,13**, CanMEDS (Health Advocate)*
Content (*What are they learning?)*	Informed consent with AI, data privacy, algorithmic bias, explainability, justice in healthcare AI, real cases/scenarios	AI-supported case simulations (e.g., robot-assisted surgery, algorithmic diagnosis of depression, GPT-generated medical notes)	CanMEDS (Health Advocate)*
Learning activities *(How are they learning?)*	Case analyses, AI policy debates/AI-assisted debates	Students defend or oppose AI’s role in clinical decisions; real-time fact checks by AI	AAMC EPA 5, 6, 10**, CanMEDS (Communicator)*
Teacher role *(How is the teacher facilitating their learning?)*	Ethics facilitator, discussion moderator, case designer	Faculty development in digital ethics facilitation	CanMEDS (Leader)*
Materials and Resources *(With what are they learning?)*	Guidelines on AI ethics, ethical frameworks, case studies related to AI use in healthcare	AI-enhanced academic search tools, AI-supported case simulations (e.g., robot-assisted surgery)	AAMC EPA 9,13**
Grouping *(With whom are they learning?)*	Peer debate teams, think-pair-share groups, interprofessional teams	Student interact with each other in groups, with medical ethicists, AI technologists, and AI itself	WHO Interprofessional Competency Framework****, CanMEDS (Collaborator)*
Location *(Where are they learning?)*	A blended learning ecosystem combining digital and physical spaces	Hybrid Learning Platforms, LMS	AAMC EPA 7**
Time *(When are they learning?)*	1-2-week module embedded in Professionalism/Ethics course	Scheduled asynchronous and synchronous digital and physical sessions, including asynchronous reflection time	CanMEDS (Professional)* (Leader -Time management)
Assessment *(How is their learning assessed?)*	Case-based essays, AI ethics position paper, participation in AI debate	Graded rubrics, faculty evaluations, Peer-assessment, self-assessments	CanMEDS (Scholar)*, AAMC EPA 7**

*CanMEDS Frameworks (76).

**Amiel et al., AAMC (77).

***AAMC, AACOM, ACGME (78).

****WHO (79).

The integration of Spider Web Model was designed to illustrate across different levels: *nano (course level), micro (module level), and meso (institutional level)*.

- *Nano* level integration stands for the incorporation of AI-related concepts and tools within individual courses, particularly in the preclinical and clinical phases. At the nano level, integration focuses on embedding AI content into existing or new courses. For example, in a course designed with this aim, students engage in case-based learning activities using AI-assisted diagnosis tools or participate in simulations where AI provides patient management recommendations. The aim is to gradually build foundational AI competencies that align with core medical learning outcomes.
- At the *micro* level, integration occurs across entire modules or thematic blocks, where AI is thematically connected to multiple disciplines. Here, AI serves as a unifying theme for interdisciplinary learning and teaching. For instance, in a Diagnostic Medicine module, students might examine how AI is used in imaging, pathology slide analysis, and predictive analytics, supported by workshops and team-based activities. AI is also explored through clinical reasoning exercises that simulate complex decision-making processes with AI tools.
- At the *meso* level, integration is strategic, systemic and institutional. AI becomes a curricular and organizational priority, reflected in institutional vision at philosophy level. Medical schools might create an “AI in Medicine” longitudinal curriculum strand, which spirals across all years of education. This multi-level approach and the holistic design offered by van den Akker’s Spider Web Model can be seen as a coherent tool to enable horizontal and vertical integration easily across the years.

All sample curriculum framework illustrations in this section have been developed using spider web components in rows, and four columns that include *explanations for the questions in the spider web model components*, *examples of implementation*, Rourke (3) *links to competency frameworks*. Table 1 presents a meso-level (institutional level) curriculum design framework that illustrates how artificial intelligence (AI) can be integrated into medical school’s curriculum using van den Akker’s Spider Web Model. This level addresses the broad vision and strategic alignment necessary for embedding AI literacy across the continuum of medical education. Each component of the spider web has been adapted to reflect institutional curriculum objectives and priorities, and future-oriented competencies expected of medical graduates. The rationale is grounded in the growing imperative to prepare future physicians for AI-assisted healthcare environments. This table was constructed to demonstrate the alignment between educational policy, curriculum structure, and competency-based medical education (CBME) principles. It is believed that it will serve as a foundational guide for policymakers and curriculum designers in defining overarching goals and ensuring consistency in AI integration at all subsequent levels of curriculum development.

Table 1 demonstrates how AI integration can be envisioned as a strategic initiative within an institutional curriculum. By systematically mapping each component of van den Akker’s Spider Web Model, the table shows that AI is not just a technical skill but a transformative domain that intersects with every aspect of medical education. The framework also emphasizes alignment with internationally recognized competency frameworks, ensuring global relevance. Overall, this design helps stakeholders (curriculum committees, accreditation bodies, and policy makers) grasp how technology and AI education can be embedded across the medical education continuum in a cohesive and future-proof manner.

Table 2 indicates a sample framework for the micro-level of curriculum design and applies the Spider Web Model to a specific module: Nervous System as an example. At this level, the framework shows how AI concepts, such as neuroimaging interpretation and brain data ethics, can be embedded directly into existing content. This enables contextualized and meaningful learning, fostering the application of AI knowledge in clinical neurology. The table maps out how AI can be intertwined with traditional learning outcomes and instructional strategies within a single module. The examples of implementation highlight the practical steps taken to ensure student engagement and curriculum coherence.

The micro-level framework in Table 2 illustrates how abstract curriculum principles from the meso level can be translated into tangible, module-specific applications. By focusing on Nervous System module, the design exemplifies how AI content can be integrated without disrupting the existing curriculum structure. The connections to specific learning activities and competencies ensure that AI is not treated as a single, individual or add-on strategy, but rather as a core enhancer of clinical reasoning and interdisciplinary understanding.

Table 3 illustrates the nano-level of curriculum design, focusing on a single course—an AI-focused Medical Ethics. This table demonstrates the application of the Spider Web Model, where each component is tailored to support ethical reasoning within the rapidly evolving landscape of AI in healthcare. The course aims to develop learners’ critical thinking and professional judgment through activities such as case-based analysis, debates, and reflective writing. Real-world case materials and internationally recognized digital ethics frameworks are used to ensure relevance and authenticity. This table exemplifies how a single course can operate complex curriculum goals in a focused, practical manner.

Table 3 provides a concrete example of curriculum design at the course level, offering a focused application of AI within the context of medical ethics. This nano-level framework shows how ethical dimensions of AI can be taught in a way that is both theoretically grounded and practically engaging. It highlights how assessment, materials, and teaching methods can be purposefully aligned to create meaningful learning experiences around digital professionalism. This level of design is particularly important for faculty members who are responsible for delivering AI content in specific courses, as it offers a clear and replicable model for curriculum implementation.

## 4 Discussion

This conceptual paper highlights the value of van den Akker’s Curricular Spider Web Model as a holistic and adaptable framework for curriculum design in medical education. By applying this model in undergraduate medical education, the paper illustrates how it can be practically used to enhance curriculum coherence and alignment. The contribution is not in creating a new theoretical model but in demonstrating the Spider Web’s utility alongside existing frameworks and curriculum development models. For example, Spider Web Model provides system-level coherence that strengthens Kern’s practical steps, enriches Harden’s integration ladder, and adds flexibility to Tyler’s objectives-based approach. The nano/micro/meso examples illustrate concrete possibilities for application, with a focus on the integration of AI across curricula.

One of the key contributions of this study lies in Spider Web Model’s complement Tyler, Kern, and Harden by giving system-level coherence in implementation. Tyler’s objective-based approach, while historically influential, has been critiqued for its linear and prescriptive nature, which may not align well with the complex, dynamic, and competency-based nature of contemporary medical curricula. Harden’s SPICES model, though innovative in promoting learner-centered and integrated strategies, primarily serves as a set of guiding principles rather than a comprehensive design framework. Kern’s Six-Step Approach, while systematic and practical, focuses mainly on course or module-level development and may not sufficiently address system-level integration.

Van den Akker’s Spider Web Model complements these frameworks by offering a theory-informed structure that visualizes curriculum as a network of interconnected elements. Its central emphasis on rationale-driven alignment that interlinks each component supports not only curricular coherence but also flexibility across nano, micro, and meso levels. This interconnectedness is particularly valuable for guiding longitudinal integration.

Theoretically, this paper contributes to literature by recontextualizing an existing model from general education and discussing its use in health professions education. It moves beyond traditional, linear, input–output models and underscores the importance of design coherence as a foundational principle. Additionally, the Spider Web Model’s metaphor effectively communicates the fragility and balance required in curriculum systems, reinforcing the view that changes to one component inevitably affect others. From a practical standpoint, the model can inform curriculum reform efforts, help institutions map their curriculum, identify misalignments, and design strategically integrated innovations. It may also support faculty development by offering a shared language and structure for curriculum planning. As stated earlier, this model could help bridge the gap between educators’ theoretical understanding and practical implementation of curriculum making.

The Spider Web Model is most valuable when curriculum challenges extend beyond single courses to systemic issues such as institutional alignment, integration of innovations, or stakeholder coherence. Therefore, for medical educators and curriculum committee members to move from conceptual understanding to actual curriculum change using the Spider Web Model, several practical issues require consideration: *governance and stakeholder engagement*, *resource planning*, *continuous faculty development*, *timelines and phasing*, and *evaluation and feedback process*.

- *Governance and stakeholder engagement*: Successful implementation requires endorsement by curriculum committees, faculty leadership, and accreditation bodies. Involving students, clinical faculty, and patient representatives in the governance structure enhances legitimacy and shared ownership.
- *Resource planning:* Redesigning curricula based on the Spider Web Model often entails investments in digital infrastructure (learning management systems, simulation labs, AI tools), teaching materials, and administrative support. Early planning of financial and human resources prevents implementation fatigue.
- *Continuous faculty development*: Faculty must be prepared not only to teach emerging content such as digital health and AI literacy but also to work with an interconnected model of curriculum design. Structured workshops, peer-learning groups, and mentorship programs can support this transition.
- *Timelines and phasing*: In curriculum development process, systemic curricular reform should be phased. Pilot implementation at the nano level (e.g., individual courses) can test feasibility before expanding to micro (module) and meso (institutional) levels. In other words, designing a specific course or module, Kern’s Six-Step Approach provides sufficient structure, however, if the aim is to develop a faculty-wide coherence plan for integrating innovations such as AI, or aligning diverse stakeholders across years, the Spider Web Model will offer better results.
- *Curriculum Evaluation and Feedback Process*: Implementation should be accompanied by formative evaluation (e.g., stakeholder feedback, curriculum mapping) and summative evaluation (e.g., student outcomes, graduate performance) to ensure ongoing alignment, and compliance with accreditation standards.

## 5 Strengths and limitations

This study offers several notable strengths. It is the first to systematically apply van den Akker’s Curricular Spider Web Model—widely used in general education—to medical education. By structuring this application with elements of Conceptual Framework Analysis (CFA), the paper provides a transparent, theory-informed approach. The inclusion of illustrative curriculum design frameworks at nano, micro, and meso levels enhances practical relevance and demonstrates flexibility across curriculum planning layers. However, the study has two limitations. First, it does not include empirical testing of the model’s implementation in real curriculum change processes. Second, in the illustrative curriculum design frameworks at nano, micro, and meso levels, mainly American and European-based competency frameworks (AAMC, CanMEDS) were referenced.

## 6 Conclusion and future directions

In an era of rapidly evolving healthcare systems and educational paradigms, medical curricula must be coherent, adaptive, and aligned across all levels. This conceptual paper introduced van den Akker’s Curricular Spider Web Model as a theoretically relevant framework for curriculum design in medical education. By highlighting the model’s interconnected structure and applying it to illustrative examples at the nano, micro, and meso levels, this study demonstrates how the Spider Web Model can complement and extend existing frameworks such as those proposed by Tyler, Harden, and Kern. It is for sure that each curriculum model presents valuable insights and tools for designing effective curriculum development process. However, Spider Web Model’s emphasis on alignment and contextual adaptability offers medical educators a valuable conceptual tool for both curriculum innovation and change. While this paper presents a theoretical adaptation, it provides a foundation for future empirical research and institutional experimentation. As medical education continues to navigate the demands of digital transformation, competency-based learning, and interdisciplinary integration, models like the Curricular Spider Web offer a promising way forward in designing coherent, learner-centered educational experiences. In addition, while this paper focuses on medical education, the Spider Web Model is equally applicable to other health professions education contexts. The shared need for integrated, competency-based curricula across these disciplines highlights the transferability and broader relevance of the model.

In the light of the results of this study, some suggestions have been made for medical educators and researchers. Spider Web Model puts emphasis on coherence and alignment across all curriculum components; therefore, curriculum implementation should no longer be viewed as a top-down process where teachers are expected to strictly follow external prescriptions. Instead, a shift toward an enactment approach is needed, where educators and learners collaboratively shape their curriculum realities. Within the Spider Web framework, this participatory approach strengthens the interconnections among key curriculum elements. Medical educators play a central role as curriculum developers, not merely implementers. Their engagement in collaborative reflection, design, and adaptation is essential to ensure that all elements of the curriculum are contextually relevant and responsive to local needs. In addition, as this paper provides a conceptual foundation for further empirical research, future studies should explore the implementation of the Spider Web Model in real-world medical education settings to evaluate its impact on curriculum alignment, faculty collaboration, and student learning outcomes. Mixed-methods approaches, including curriculum mapping, interviews with faculty, and analysis of student performance data, can be used to assess the model’s practical utility.

## Data Availability

The original contributions presented in the study are included in the article/supplementary material, further inquiries can be directed to the corresponding author.
